# Microbial spectrum and antimicrobial resistance are associated with outcomes after endoscopic ultrasound-guided therapy of pancreatic fluid collections

**DOI:** 10.1038/s41598-026-64436-5

**Published:** 2026-08-05

**Authors:** Marcel Razpotnik, Christian Jürgensen, Michael Sigal, Toni Herta, Özlem Çelebi, Andreas Adler, Silke Leonhardt, Federica Branchi, Juliane Buchkremer, Konstantinos Kouladouros, Alexander Wree, Jürgen Pilz, Markus M. Heimesaat, Britta Siegmund, Cornelius Engelmann, Frank Tacke

**Affiliations:** 1 Department of Hepatology and Gastroenterology, Campus Virchow- Klinikum (CVK) and Campus Charité Mitte (CCM), Charité - Universitätsmedizin Berlin, Berlin, Germany; 2 Department of Gastroenterology, Infectology and Rheumatology, Campus Benjamin Franklin (CBF), Charité - Universitätsmedizin Berlin, Berlin, Germany; 3 Department of Gastroenterology, Vivantes Hospital Friedrichshain Berlin, Berlin, Germany; 4 Department of Statistics, University of Klagenfurt, Klagenfurt, Austria; 5 Institute of Microbiology, Infectious Diseases and Immunology, Charité - Universitätsmedizin Berlin, Corporate Member of Freie Universität Berlin, Humboldt-Universität zu Berlin, Berlin Institute of Health, Berlin, Germany

**Keywords:** Pancreatic necrosis, Walled-off necrosis, Pancreatic pseudocyst, Multidrug-resistant, Enterococcus infection, Endoscopic ultrasound-guided drainage, Necrosectomy, Diseases, Gastroenterology, Medical research, Microbiology

## Abstract

Infected pancreatic fluid collections (PFC) are among the most challenging entities in therapeutic endoscopy, requiring multiple interventions. In this multicenter study, we investigated whether microbiological profiles and antimicrobial resistance are associated with outcomes after endoscopic ultrasound-guided drainage. The primary endpoint was time to clinical success, defined as radiographic resolution of the PFC to < 30 mm, no need for additional intervention, and clinical improvement. Secondary outcomes included surgery-free survival, number of interventions, and ICU stay. Among 398 included patients (median age: 56.5 years; 70.6% male), 232 (58.3%) had microbiologically confirmed infection. Complete resolution was achieved in 89.2% of infected PFCs (median: 70 days; IQR 37–118) versus 95.2% of sterile PFCs (57 days; IQR 33–91). Resolution was significantly delayed in *Enterococcus* (*n* = 107; 26.9%) and polymicrobial resistant infections (*n* = 38; 9.5%): 87 days (*p* = 0.003) and 120 days (*p* < 0.001), respectively. *Enterococcus* species remained an independent predictor in multivariable Cox regression, and in a sensitivity analysis restricted to the necrosectomy group (aHR 2.50; *p* = 0.002). Vancomycin-resistant *Enterococcus* (VRE) infection (*n* = 26; 6.5%) was associated with the highest number of interventions (median: 6), longest ICU stays (median: 72 days), and highest mortality (23%). In summary, this study identifies an association of *Enterococcus* species and antimicrobial resistance with poor outcomes after EUS-guided therapy, emphasizing the importance of microbiology-informed treatment strategies.

**Supplementary Information:**

The online version contains supplementary material available at 10.1038/s41598-026-64436-5.

## Introduction

Pancreatic and peripancreatic fluid collections (PFCs) are among the most significant and challenging complications of pancreatitis^1^. They may also arise following abdominal trauma or biliopancreatic surgery and often require multidisciplinary interventional management^2,3^. Current therapeutic strategies are primarily based on imaging criteria, such as encapsulation, necrosis, size, and paracolic extension^4^.

In recent years, PFC management has evolved toward individualized, minimally invasive approaches, including multigateway and multimodal techniques. If endoscopic ultrasound (EUS)-guided drainage alone does not lead to clinical improvement, transluminal necrosectomy is the preferred next step^5^. An endoscopic step-up approach is preferred over surgery due to fewer complications, including pancreatic fistula^6^; upfront endoscopic necrosectomy may even accelerate time to clinical success^7^.

Timely and effective drainage is particularly critical in infected PFCs to prevent sepsis and related complications. However, endoscopic intervention itself may introduce infection into a previously sterile collection, potentially resulting in severe or even fatal outcomes^8^.

The widespread use of antibiotics in recent decades has shifted the microbial spectrum from Gram-negative to Gram-positive bacteria—particularly *Staphylococcus* and *Enterococcus* species—along with increased rates of fungal and drug-resistant infections^9,10^. These microorganisms have been linked to higher mortality in infected pancreatic necrosis^11^.

Despite the widespread adoption of interventional endoscopy, the impact of microbial profiles on outcomes following EUS-guided therapy remains poorly understood, representing an important knowledge gap with direct implications for therapeutic decision-making and resource allocation.

We hypothesize that treatment strategies should extend beyond imaging criteria to incorporate microbiological profiles and infection complexity, which may significantly impact clinical outcomes. This study aimed to explore the relationship between the microbial spectrum, multidrug resistance, and the efficacy of EUS-guided therapy for infected PFCs.

## Methods

### Study design and data source

This multicenter retrospective cohort study was conducted between January 2017 and December 2023 at three tertiary referral centers within Charité -Universitätsmedizin Berlin: Campus Virchow-Klinikum, Campus Charité Mitte, and Campus Benjamin Franklin. Consecutive patients with encapsulated PFCs, irrespective of etiology, who underwent EUS-guided drainage were screened for eligibility. Pancreatitis-associated collections were classified according to the revised Atlanta classification^12^.

Exclusion criteria included age < 18 years, pregnancy, prior PFC drainage (including external procedures before referral), collections located > 1 cm from or not adherent to the gastrointestinal wall, endoscopically inaccessible collections, concurrent EUS-guided hepatobiliary-pancreatic interventions, severe coagulopathy (INR > 1.5), thrombocytopenia (platelet count < 50,000/mm³), incomplete clinical data, or lack of follow-up information (Supplementary Fig. 1).

All patients or their legally authorized representatives provided written informed consent. The study was conducted according to the principles of the 2013 Declaration of Helsinki and approved by the institutional review board of Charité-Universitätsmedizin Berlin (approval number EA4/041/24).

### Study outcomes and definitions

The primary outcome was time to clinical success, defined as radiological reduction of the peripancreatic fluid collection to < 30 mm in diameter on cross-sectional imaging, no need for additional intervention, and clinical improvement^13^. Secondary outcomes included surgery-free survival, length of hospital and intensive care unit (ICU) stay, and the total number of interventions required to achieve treatment success, including the initial procedure, stent removal or replacement, and additional interventions such as necrosectomy or complication management.

### Follow-up evaluation

Patients underwent surveillance for 12 months following the index procedure. Standardized cross-sectional imaging with contrast-enhanced computed tomography or magnetic resonance imaging was performed at baseline and continued until clinical success was documented. Imaging parameters were assessed at predetermined intervals: baseline (pre-intervention), early post-intervention (within 30 days), intermediate (31–90 days), and delayed (91–365 days).

Resolution of the collection was assessed by comparing the maximal diameter on follow-up imaging with baseline measurements. All imaging studies were independently reviewed by a consultant radiologist and a senior gastroenterologist with pancreatic expertise, each blinded to the infection status of the PFC, using standardized measurement protocols with consistent reference planes. For morphologically complex collections with irregular contours, standardized measurements across follow-up imaging enabled longitudinal assessment of dynamic changes rather than reliance on isolated absolute values, enhancing measurement reliability.

### Endosonographic intervention and treatment algorithm

All procedures were performed or supervised by experienced interventional endoscopists defined as having independently performed > 25 EUS-guided drainage procedures, using linear echoendoscopes under monitored deep sedation or general anesthesia^14^. Prophylactic antibiotic therapy was administered at the endoscopist’s discretion.

Following comprehensive endosonographic assessment and color Doppler evaluation to identify optimal access sites, collections were punctured using a 19-gauge fine-needle aspiration (FNA) needle. Aspirated fluid was transferred under sterile conditions into microbiological transport media for culture and antimicrobial susceptibility testing.

Lumen-apposing metal stents (LAMS) and double-pigtail plastic stents (DPS) were used for drainage; in necrotic collections, at least three plastic stents were placed.

In patients without adequate clinical improvement within 3–5 days, treatment was escalated according to an endoscopic step-up approach, including stent optimization or replacement, multigateway drainage, percutaneous catheter drainage, or transluminal necrosectomy for infected necrosis. Necrosectomy was performed using a standard-diameter gastroscope and repeated at 3- to 5-day intervals until near-complete endoscopic debridement was achieved.

LAMS were generally removed within 4–6 weeks after placement and exchanged for DPS when prolonged drainage was required but further necrosectomy was no longer indicated.

### Microbiological assessment

Infected PFCs were diagnosed based on positive cultures obtained at index drainage or from pre-drainage aspirates, together with elevated inflammatory markers and clinical evidence of infection, including fever, clinical deterioration, or lack of improvement^15^. Alternatively, positive blood cultures were considered diagnostic after exclusion of extra-pancreatic infection sources, including pneumonia, urinary tract infection, and cholangitis.

Previous antibiotic therapy was defined as systemic antibiotic treatment administered within 14 days before or on the day of the index procedure. Polymicrobial infection was defined as isolation of ≥ 2 distinct microbial species from a single specimen. Bacteria were classified according to antibiotic susceptibility profiles as defined by the European Committee for Antimicrobial Susceptibility Testing (EUCAST)^16^(Supplementary Material). Inadequate antimicrobial therapy (IAT) was defined as systemic antimicrobial treatment lacking in vitro activity against at least one of the identified pathogens, based on susceptibility testing.

### Statistical analysis

Normality of continuous variables was assessed using the Shapiro-Wilk test. Data are presented as mean ± standard deviation, median with interquartile range (IQR), or number and percentage. Group comparisons employed Student’s t-test for normally distributed variables and the Kruskal-Wallis test for non-parametric data. To control for multiple exploratory baseline comparisons, a Bonferroni correction was applied, setting the adjusted significance threshold at *p* < 0.001 (0.05/54).

Time-to-event outcomes were analyzed using Kaplan-Meier estimates and compared using the log-rank test. Univariable Cox proportional hazards regression was performed to identify potential predictors of incomplete resolution and surgery-free survival, reported as hazard ratios (HRs) with 95% confidence intervals (CIs).

Multivariable models included clinically relevant variables identified through literature review and univariable screening, adjusting for age, sex, comorbidity, collection size, PFC etiology, necrosis extent, and polymicrobial infection. Variables exhibiting strong collinearity or conceptual overlap were not included simultaneously to ensure regression stability.

To address confounding, risk factors for PFC infection were assessed using multinomial logistic regression, comparing *Enterococcus* species, non-*Enterococcus* species, and sterile controls. Results are expressed as relative risk ratios (RRRs) with 95% CIs.

The robustness of microbiological determinants across procedural complexity strata was assessed through a sensitivity analysis restricted to patients undergoing endoscopic necrosectomy, adjusting for identified risk factors. Statistical significance was set at *p* < 0.05 unless otherwise specified. Analyses were performed using IBM SPSS Statistics (Version 29.0.0.0).

## Results

### Cohort Characteristics and Baseline Overview

Among 517 consecutive patients with PFCs who underwent EUS-guided intervention, 398 met the eligibility criteria (232 infected, 166 non-infected) and were included in the final analysis (Supplementary Fig. 1). The median age was 56.5 years (IQR 46–65 years), with a male predominance (70.6%). During the 12-month follow-up, 1,227 endoscopic procedures were performed and 693 imaging studies reviewed, with quantitative assessment of fluid collections in each case. The median interval from symptom onset to initial drainage was 21 days (IQR 12–33 days). Endoscopic transmural necrosectomy was performed in 30.9% of patients, accounting for 366 procedures. Microbiological data from fine-needle aspirates were available in 330 (82.9%), and blood cultures in 261 (65.6%).

Infection was the most common indication for EUS-guided drainage, present in 232 of 398 patients (58.3%), followed by mass effect-related symptoms in 94 patients (23.6%), including pain, gastric outlet obstruction, or biliary obstruction with jaundice. The remaining 72 patients (18.1%) were asymptomatic and had no clinical signs of infection but showed progressive PFC enlargement on interval imaging.

Baseline characteristics are summarized in Table 1.

**Table 1:** Baseline characteristics.

	All patients	Non- infected	Infected	*p*-value^a^
	(*n* = 398)	(*n* = 166)	(*n* = 232)	
**Patient demographics**				
Age (years)	56.5 (46.0–65.0)	57.5 (46.0–66.0)	56.0 (46.5–63.0)	0.60
Male sex (%)	281 (70.6)	107 (64.5)	174 (75)	0.03
BMI (kg/m²)	24.4 (21.3–28.7)	23.8 (20.9–28.3)	24.8 (21.6–29.0)	0.27
**Comorbidities (%)**	288 (72.4)	123 (74.1)	165 (71.1)	0.59
Cardiac (%)	99 (24.9)	43 (25.9)	56 (24.1)	0.77
Pulmonary (%)	94 (23.6)	33 (19.9)	61 (26.3)	0.17
Renal (%)	51 (12.8)	13 (7.8)	38 (16.4)	0.02
Hepatic (%)	58 (14.6)	12 (7.2)	46 (19.8)	0.0007
Others (%)	195 (49.0)	92 (55.4)	103 (44.4)	0.04
ASA classification				
1 (%)	43 (10.8)	16 (9.6)	27 (11.6)	0.64
2 (%)	154 (38.7)	78 (46.9)	76 (32.8)	0.006
≥ 3 (%)	201 (50.5)	72 (43.4)	129 (55.6)	0.02
Pre-drainage organ failure (%)	72 (18.1)	12 (7.2)	60 (25.9)	< 0.0001
SIRS (%)	32 (8.0)	3 (1.8)	29 (12.5)	0.0006
Previous antibiotic therapy (%)	278 (69.8)	104 (62.7)	174 (75)	0.01
Immunosuppressive therapy (%)	37 (9.3)	15 (9.0)	22 (9.5)	0.97
**Etiology**				
Acute pancreatitis (%)	207 (52.0)	67 (40.4)	140 (60.3)	0.0001
Chronic pancreatitis (%)	43 (10.8)	24 (14.5)	19 (8.2)	0.07
Post-pancreatectomy (%)	148 (37.2)	75 (45.2)	73 (31.5)	0.007
**Laboratory**				
Hemoglobin (g/dL)	10.1 (8.7–11.7)	10.8 (9.3–12.6)	9.6 (8.5–11.1)	< 0.0001
Sodium (mmol/L)	138 (136–140)	139 (136–141)	138 (135–140)	0.02
C-reactive protein (mg/L)	91.7 (30.4–179)	62 (24–118)	119 (44.3–205)	< 0.0001
Procalcitonin (µg/L)	0.29 (0.12–0.75)	0.13 (0.04–0.33)	0.40 (0.17–0.87)	0.0001
Creatinine (mg/dL)	0.74 (0.58–0.96)	0.74 (0.59–0.88)	0.73 (0.57–1.00)	0.64
Lipase (IU/L)	40.0 (20.0–85.0)	55 (24–100)	37 (18.8–67.3)	0.01
Quick (%)	82.0 (70.0–95.0)	87.5 (77–100)	78.0 (66–91)	< 0.0001
**PFC characteristics**				
Location of PFC				
Head (%)	134 (33.7)	46 (27.7)	88 (37.9)	0.04
Body (%)	179 (45.0)	64 (38.6)	115 (49.6)	0.04
Tail (%)	197 (49.5)	74 (44.6)	123 (53.0)	0.13
Presence of necrosis (%)	164 (41.2)	46 (27.7)	118 (50.9)	< 0.0001
Necrosis > 30% (%)	61 (15.3)	14 (8.4)	47 (20.3)	0.002
Extension to paracolic gutter (%)	97 (24.4)	18 (10.8)	79 (34.1)	< 0.0001
Encapsulated PFC				
Fully (%)	316 (79.4)	130 (78.3)	186 (80.2)	0.74
Partially (%)	82 (20.6)	36 (21.7)	46 (19.8)	0.74
Size of PFC (mm)	80 (56–108)	70 (47–95.5)	89 (60–119)	< 0.0001
Size > 8 cm (%)	191 (48.0)	58 (34.9)	133 (57.3)	< 0.0001
Recurrence rate (%)	43 (10.8)	12 (7.2)	31 (13.4)	0.07
Time to recurrence (days)	80 (44.3–173)	63.5 (34.0–116)	140 (45.0–196)	0.27
**Intervention characteristics**				
Time from onset to drainage (days)	21 (12.0–33.0)	17 (10.0–29.5)	23.5 (14.0–34.5)	0.01
Route of EUS-guided drainage				
Transgastric (%)	371 (93.2)	155 (93.4)	216 (93.1)	0.93
Transduodenal (%)	22 (5.5)	9 (5.4)	13 (5.6)	0.89
Both (%)	5 (1.3)	2 (1.2)	3 (1.3)	0.71
Type of stent used				
Double pigtail stent (%)	343 (86.2)	137 (82.5)	206 (88.8)	0.10
LAMS (%)	55 (13.8)	29 (17.5)	26 (11.2)	0.10
Stent dislocation (%)	60 (15.1)	24 (14.5)	36 (15.5)	0.89
Time to stent removal (days)	70 (53.8–98)	66 (50–93.8)	73 (52–101)	0.29
Endoscopic necrosectomy^b^ (%)	123 (30.9)	29 (17.5)	94 (40.5)	< 0.0001
Percutaneous drainage^b^ (%)	59 (14.8)	15 (9.0)	44 (19.0)	0.009
Laparoscopic necrosectomy^b^ (%)	13 (3.3)	2 (1.2)	11 (4.7)	0.10
Surgical intervention^b^ (%)	22 (5.5)	2 (1.2)	20 (8.6)	0.003
ERCP before drainage (%)	99 (24.9)	32 (19.3)	67 (28.9)	0.04
ERCP within 30 days (%)	38 (9.5)	12 (7.2)	26 (11.2)	0.24
Pancreatic stent (%)	39 (9.8)	15 (9.0)	24 (10.3)	0.80
Disconnected pancreatic duct (%)	25 (6.3)	12 (7.2)	13 (5.6)	0.66

Abbreviations: ASA, American Society of Anesthesiologists; BMI, body mass index; ERCP, endoscopic retrograde cholangiopancreatography; EUS, endoscopic ultrasound; LAMS, lumen-apposing metal stent; PFC, pancreatic fluid collection; SIRS, systemic inflammatory response syndrome; ^a^Adjusted significance threshold after Bonferroni correction: *p* < 0.001; ^b^Step-up multimodal therapy following initial endoscopic drainage.

### The spectrum of microorganisms and antimicrobial susceptibility profiles

**Fig. 1:**
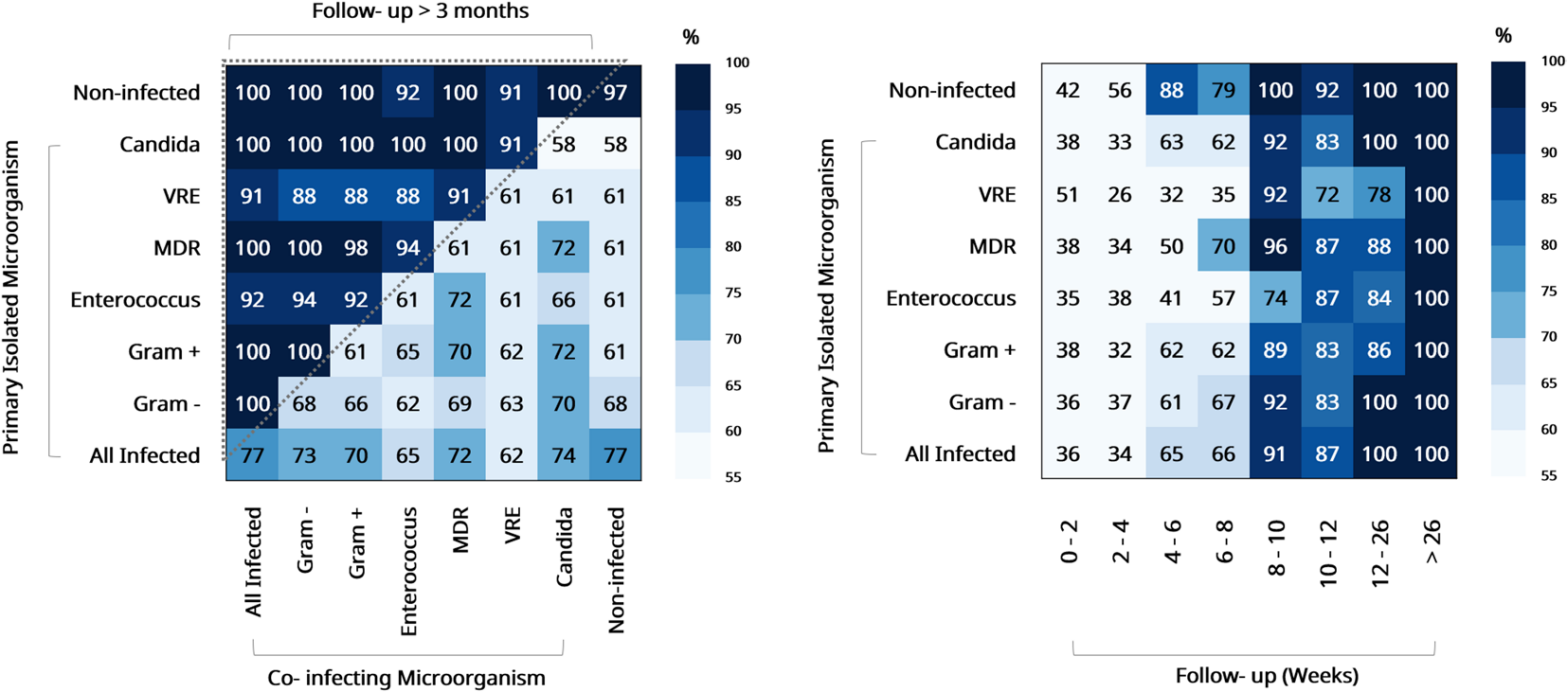
Resolution rates stratified by microbial combinations and time after the initial EUS-guided intervention. Abbreviations: MDR, multidrug-resistant; VRE, vancomycin-resistant *Enterococcus*. Note (coinfection diagram): The left-facing triangle indicates resolution rates beyond 3 months of follow-up; the right-facing triangle indicates resolution rates within 3 months. Each box represents the median value for the corresponding X–Y determinant combination.

Among 502 microbiological reports, at least one positive culture was identified in 232 patients, while 270 showed no microbial growth. Nearly half of the positive cases were polymicrobial (median: 3 microorganisms; IQR 2–4) (Fig. 1). Previous or concurrent antimicrobial therapy was administered in 312 patients (78.4%), including 104 (62.7%) with presumed sterile collections who received empirical prophylaxis. The most frequently administered agents were penicillin derivatives (42%), meropenem (38.2%), vancomycin (21.4%), cephalosporins (20.1%), and antifungals (17.8%), mostly in combination (median: 2 agents; IQR 1–3). The median treatment duration was 14 days (IQR 9–30) (Supplementary Table 1). Initial inadequate antimicrobial therapy requiring subsequent modification was observed in 70 cases (30.2%).

The most prevalent bacterial families were *Enterococcaceae* (26.9%), *Streptococcaceae* (13.1%), and *Staphylococcaceae* (9.8%). Gram-negative species were less common, with *Escherichia coli* (8.3%) and *Klebsiella pneumoniae* (7.3%) being the most prevalent. Among fungal isolates, *Candida albicans* was most frequently identified (21.9%), followed by *Candida glabrata* (6.3%) (Table 2, Supplementary Table 2).

Positive bacterial cultures were categorized by antimicrobial susceptibility profiles. Multidrug resistance was identified in 29.6% of infected patients, with 1.5% demonstrating extensive drug resistance and one case of panresistance. VRE, multidrug-resistant Gram-negative bacilli (MRGN), and methicillin-resistant *Staphylococcus aureus* (MRSA) were isolated in 26, 15, and 3 patients, respectively.

### Microbial spectrum and antimicrobial resistance are associated with delayed resolution and poorer clinical outcomes following EUS-guided therapy

During follow-up, 207 of 232 infected collections (89.2%) resolved on imaging, fewer than in the control group (158 of 166 [95.2%]; χ² test, *p* = 0.05). Median time to resolution was longer in the infected group (70 days; IQR 37–118) compared with controls (57 days; IQR 33–91), with borderline significance (log-rank *p* = 0.08). Clinical success did not differ by stent type (LAMS 90.9% vs. DPS 91.8%; *p* = 0.96), and treatment era was not associated with clinical success (2021–2023 vs. 2017–2020: HR 0.84, 95% CI 0.66–1.06; *p* = 0.15).

**Fig. 2:**
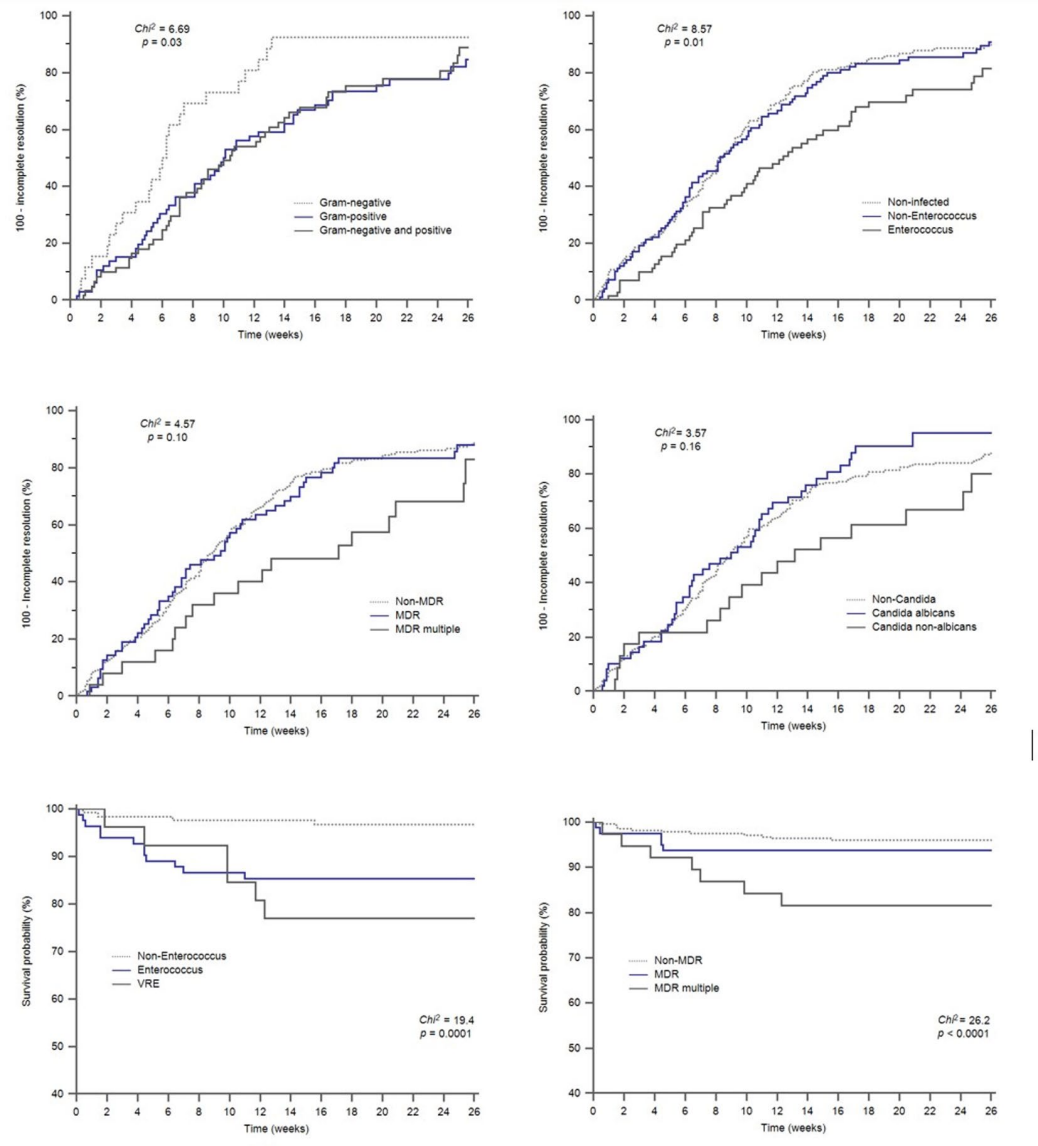
Cumulative incidence of clinical failure and surgery-free survival stratified by infecting organism and antimicrobial resistance. Abbreviations: MDR, multidrug-resistant; VRE, vancomycin-resistant *Enterococcus*. Note: p-values are based on the log-rank test.

Gram-negative bacterial infections were associated with high clinical success rates (89.4%) and shorter resolution time (median 61 days; IQR 36–105), comparable to non-infected PFCs. In contrast, Gram-positive infections—particularly *Enterococcus* species—were associated with significantly worse outcomes, including a longer median time to resolution (87 days; IQR 46–173; log-rank *p* = 0.003) (Fig. 2). *Enterococcus* infections were also linked to prolonged hospital stay (45 days; IQR 23–83; *p* < 0.001) and greater intensive care utilization (16 days; IQR 5–56; *p* < 0.001) compared with other infections.

Reinterventions were notably frequent in patients with VRE infections (median 6; IQR 3–13.8) and polymicrobial infections involving multiple MDRs (median 6; IQR 3–11.3). Both groups demonstrated the least favorable clinical outcomes among all infecting microorganisms, with resolution rates of 73.1% and 76.3%, respectively, and median resolution times approaching 120 days (Table 2).

### Multivariable analysis identifies an association between *Enterococcus* species and incomplete resolution

In multivariable Cox regression analysis, collection diameter > 8 cm (aHR 1.33, 95% CI 1.00–1.79; *p* = 0.05) and pancreatitis as the underlying etiology (aHR 1.37, 95% CI 1.03–1.82, *p* = 0.03) emerged as independent predictors of incomplete radiographic resolution.

Among microbiological determinants, *Enterococcus* species were independently linked to incomplete resolution (aHR 1.47, 95% CI 1.03–2.13; *p* = 0.03), driven predominantly by *Enterococcus faecium* (aHR 1.67, 95% CI 1.02–2.73; *p* = 0.04), whereas *Enterococcus faecalis* showed no significant association (aHR 1.59, 95% CI 0.79–3.19; *p* = 0.19). The association between *Enterococcus* species and incomplete resolution strengthened in an analysis restricted to infected walled-off necrosis (aHR 2.24, 95% CI 1.37–3.67; *p* = 0.001) (Table 4, Supplementary Table 3).

Collections harboring multiple multidrug-resistant organisms demonstrated a trend toward an increased risk of incomplete resolution (aHR 1.56, 95% CI 0.96–2.56), with borderline statistical significance (*p* = 0.07).

Demographic variables, including age ≥ 65 years, male sex, and ASA ≥ 3, as well as organ failure prior to drainage, presence of any amount of necrosis, and stent type, were not independently associated with clinical outcomes.

### Colonization with *Enterococcus* species and Vancomycin-resistant bacteria is associated with reduced surgery-free survival

During follow-up, 27 patients (6.8%) died, excluding three whose deaths were attributable to the progression of unrelated malignancy. Additionally, 22 patients (5.5%) required surgical intervention, including 13 (3.3%) who underwent laparoscopic necrosectomy. In total, 47 patients (11.8%) either died or required surgery and were included in the composite endpoint analysis.

Multivariable analysis demonstrated that infected PFCs were independently associated with reduced surgery-free survival (aHR 6.95, 95% CI 2.13–22.6), with the strongest effect observed in collections harboring *Enterococcus* species. Mortality was highest among patients with VRE infection (23.1%), significantly higher than that in other infected collections (8.3%; log-rank *p* = 0.02) and other Gram-positive infections (9.0%; log-rank *p* = 0.005).

### Sensitivity analysis in the subgroup of patients undergoing necrosectomy

A sensitivity analysis was conducted in the step-up therapy group undergoing necrosectomy to assess the consistency of microbiological predictors across procedural complexity levels. *Enterococcus* infection was associated with underlying necrosis and the need for a step-up approach, including transluminal necrosectomy (RRR 5.0, 95% CI 2.88–8.67; *p* < 0.001) (Supplementary Tables 4 and 5). Patients requiring necrosectomy presented with significantly larger collections (OR 3.05, 95% CI 1.67–5.57; *p* < 0.001) and were more likely to have acute pancreatitis as the underlying etiology (OR 3.08, 95% CI 1.55–6.12; *p* = 0.001).

In this subgroup, *Enterococcus* species remained strongly associated with incomplete resolution (aHR 2.50, 95% CI 1.39–4.55; *p* = 0.002), with the effect magnitude substantially larger than in the overall cohort. Gram-positive bacteria (aHR 2.08, 95% CI 0.89–5.00; *p* = 0.09) and non-*albicans Candida* species (aHR 1.61, 95% CI 0.88–2.94; *p* = 0.12) demonstrated trends toward delayed resolution that did not achieve statistical significance (Tables 3 and 4, and 5).

**Table 2:** Clinical outcomes stratified by infecting microorganism and antimicrobial resistance profile.

		Clinical success	Median time^c^	Hospital stay	ICU stay	Interventions	1-Y Mort^d^	Surg/Mort^e^
	% (*N*)	% (*N*)	days (IQR)	days (IQR)	days (IQR)	*N* (IQR)	% (*N*)	% (*N*)
**Non-infected**	41.7 (166)	95.2 (158)	57 (33–91)	16 (8–30)	2 (1–9)	3 (2–4)	1.2 (2)	2.4 (4)
**Infected**	58.3 (232)	89.2 (207)	70 (37–118)	29 (15–54)^b^	9 (2–38)^b^	4 (2–7)^b^	9.5 (22)^a^	18.5 (43)^b^
**Gram-positive**	45.5 (181)	86.7 (157)^a^	71 (40–140)^a^	32 (18–63.5)^b^	12 (3–48)^b^	4 (2–8)^b^	10.5 (19)^b^	18.8 (34)^b^
**Gram-negative**	30.9 (123)	89.4 (110)	61 (36–105)	29 (15–55.5)^b^	8 (2–41.8)^a^	4 (2–7)^a^	9.8 (12)^a^	19.5 (24)^b^
**Polymicrobial**	49.7 (198)	88.4 (175)^a^	68 (37–118)	31 (18–59)^b^	11 (2–41)^b^	4 (2–7)^b^	10.1 (20)^b^	18.2 (36)^b^
**Staphylococcus spp.**	9.8 (39)	89.7 (35)	53 (34–117)	33 (19.8–59.5)^b^	6 (2–32)^a^	2.5 (2–8)	12.8 (5)^a^	20.5 (8)^b^
**Streptococcus spp.**	13.1 (52)	94.2 (49)	68 (44–98)	19 (8.3–28.8)	2 (2–12.3)	4 (2–6)^a^	0	3.8 (2)
**Enterococcus spp.** ^**f**^	26.9 (107)	80.4 (86)^b^	87 (46–173)^a^	45 (23–83)^b^	16 (5–56)^b^	4 (2–9)^b^	16.8 (18)^b^	28.0 (30)^b^
**Escherichia coli**	8.3 (33)	90.9 (30)	46 (28–91)	35 (16.8–66.8)^b^	13.5 (3–46)	4 (2–6)	6.1 (2)	18.2 (6)^b^
**Klebsiella pneumoniae**	7.3 (29)	93.1 (27)	85 (50–95)	21 (12–62.8)^a^	11.5 (2–48)^a^	3 (2.5–6.5)^a^	10.3 (3)^a^	17.2 (5)^a^
**Candida albicans**	21.9 (87)	88.5 (77)	72 (34–113)	35 (19.8–62.5)^b^	12 (4–34.5)^b^	3 (2–6.3)^a^	10.3 (9)^a^	19.5 (17)^b^
**Candida**,** non-albicans**	12.6 (50)	78.0 (39)^b^	60 (31–102)	46 (25–83)^b^	12 (4–38)^b^	4 (2–9)^a^	12 (6)^a^	22 (11)^b^
**MDR**	29.6 (118)	87.3 (103)^a^	69 (36–120)	38.5 (21–75)^b^	13 (4–50.5)^b^	4 (2–8)^b^	9.3 (11)^a^	18.6 (22)^b^
**Multiple MDR **	9.5 (38)	76.3 (29)^b^	120 (50–178)^a^	51 (19–107)^b^	48 (7.8–86.3)^b^	6 (3–11.3)^b^	18.4 (7)^b^	34.2 (13)^b^
**XDR**	1.5 (6)	100 (6)	77 (18–82)	35 (15–89)^a^	34.5 (8.5–116)^a^	6 (2–9)^a^	16.7 (1)	66.7 (4) ^b^
**VRE** ^**g**^	6.5 (26)	73.1 (19)^a^	73 (50–178)	63.5 (29–129)^b^	72 (8.5–100.3)^b^	6 (3–13.8)^b^	23.1 (6)^b^	38.5 (10)^b^
**MRGN**	3.8 (15)	80.0 (12)	75 (21–251)	88 (36–142.3)^b^	58 (16.5–99.5)^b^	5 (3–9.8)^a^	20 (3)^b^	33.3 (5)^b^

Abbreviations: ICU, intensive care unit; IQR, interquartile range; MDR, multidrug-resistant; MRGN, multidrug-resistant Gram-negative bacteria; N, number; VRE, vancomycin-resistant Enterococcus; XDR, extensively drug-resistant. Compared with the control group (non-infected pancreatic fluid collections): ^a^*p*<0.05, ^b^*p*<0.001; ^c^Median time to clinical success compared by log-rank test; ^d^1-year mortality; ^e^Composite outcome of surgery and death within 1 year of the index procedure; ^f^ Including VRE cases; ^g^23 out of 26 (88.5%) vancomycin-resistant *Enterococcus faecium*.

**Table 3:** Univariable Cox regression analysis identified predictors of incomplete PFC resolution, with sensitivity analysis performed in the necrosectomy subgroup.

Variables	All PFCs		PFCs with Necrosectomy	
Patient characteristics	HR (95% CI)	*p*-value	HR (95% CI)	*p*-value
Age ≥ 65 years	0.99 (0.75–1.30)	0.94	0.66 (0.37–1.19)	0.17
Male sex	1.30 (1.01–1.67)	0.05	1.27 (0.74–2.17)	0.39
BMI ≥ 30 kg/m²	0.83 (0.59–1.18)	0.29	0.53 (0.27–1.03)	0.06
Comorbidity	1.16 (0.90–1.52)	0.25	1.25 (0.75–2.08)	0.40
ASA ≥ 3	1.22 (0.96–1.54)	0.10	1.45 (0.92–2.33)	0.11
Pre-drainage organ failure	1.01 (0.75–1.35)	0.95	0.68 (0.38–1.20)	0.19
**Collection characteristics**				
Size > 8 cm	1.54 (1.20–1.96)	< 0.001	2.44 (1.47–4.17)	< 0.001
Etiology	1.56 (1.22–2.00)	< 0.001	1.47 (0.81–2.70)	0.21
Presence of necrosis	1.35 (1.05–1.72)	0.02	1.59 (0.72–3.45)	0.25
Necrosis extent > 30%	1.20 (0.79–1.82)	0.37	1.12 (0.67–1.85)	0.67
**Therapy**				
DPS	0.76 (0.52–1.10)	0.15	0.53 (0.28–0.98)	0.04
Multiple stents	1.04 (0.81–1.33)	0.76	0.40 (0.23–0.69)	0.001
Percutaneous drainage	0.94 (0.74–1.20)	0.63	1.75 (0.91–3.33)	0.09
Immunosuppressive therapy	0.85 (0.57–1.25)	0.40	0.71 (0.28–1.76)	0.46
Previous antibiotic therapy	1.10 (0.85–1.41)	0.48	1.40 (0.64–3.06)	0.41
IAT	1.28 (0.89–1.86)	0.17	1.71 (0.95–3.06)	0.07
**Infection characteristics**				
Infection	1.23 (0.97–1.56)	0.09	1.43 (0.84–2.44)	0.19
Gram-positive	1.30 (1.02–1.67)	0.03	1.85 (1.15–3.03)	0.01
Gram-negative	1.01 (0.78–1.32)	0.95	0.90 (0.56–1.43)	0.66
Polymicrobial	1.14 (0.89–1.43)	0.30	1.35 (0.82–2.22)	0.24
*Enterococcus spp.*	1.56 (1.16–2.08)	0.004	2.33 (1.43–3.70)	< 0.001
VRE	1.49 (0.88–2.56)	0.13	1.37 (0.71–2.63)	0.35
MDR	1.15 (0.88–1.49)	0.32	1.49 (0.93–2.38)	0.09
Multiple MDR	1.67 (1.04–2.63)	0.03	1.41 (0.78–2.56)	0.26
XDR	1.19 (0.91–1.56)	0.21	1.56 (0.98–2.50)	0.06
MRGN	1.35 (0.67–2.70)	0.40	1.18 (0.53–2.56)	0.69
*Candida albicans*	0.92 (0.68–1.23)	0.55	0.79 (0.47–1.30)	0.35
*Candida*, non-albicans	1.39 (0.95–2.04)	0.09	1.75 (1.01–3.13)	0.04

ASA, American Society of Anesthesiologists; BMI, body mass index; DPS, double-pigtail plastic stents; HR, hazard ratio; IAT, inadequate antimicrobial therapy; MDR, multidrug-resistant; MRGN, multi resistant Gram-negative bacteria; PFC, pancreatic fluid collection; VRE, vancomycin-resistant Enterococcus; XDR, extensively drug-resistant.

## Discussion

In this large multicenter real-world cohort, *Enterococcus* infection was independently associated with incomplete PFC resolution and a significantly longer time to clinical success after EUS-guided therapy than that observed in sterile collections (87 days vs. 57 days; log-rank *p* = 0.003). The longest resolution time was observed in polymicrobial resistant infections (120 days; log-rank *p* < 0.001). Notably, the VRE group was linked to the highest number of interventions (median: 6) and the longest ICU stays (median: 72 days), which were eight times longer than those in other infected PFCs. VRE infections were also associated with the highest mortality rate, exceeding 23%.

Previous risk-stratification approaches have focused primarily on disease morphology. Baroud and colleagues classified patients according to the extent of necrosis, distribution across abdominal quadrants, and presence of infection. Patients with more extensive disease had a longer time to symptomatic resolution (79.6 days vs. 48.4 days; *p* = 0.02), numerically higher mortality (15% vs. 0%; *p* = 0.18), and greater need for overall endoscopies (median: 4 vs. 2; *p* = 0.001)^13^. Although infected walled-off necrosis was associated with poorer outcomes, that study did not distinguish between microbial species, resistance patterns, or the complexity of the infection.

Radiological resolution is not uniformly defined in the literature, with previous studies applying a reduction in collection diameter of > 50% or other size-based thresholds^17,18^. We therefore used the criteria of the aforementioned risk-stratification system to maintain consistency with established terminology and outcome reporting.

Similarly, Chandrasekhara et al. evaluated predictors of endoscopic step-up therapy in patients with PFCs irrespective of etiology. Collection size, paracolic extension, and necrosis exceeding 30% were identified as independent predictors of reintervention following EUS-guided drainage in multivariable logistic regression analysis and remained significant even after excluding postoperative collections. Complete resolution was achieved in 94.9% of patients after a median of 64 days, closely aligning with our findings (92% resolution; median 63 days)^19^.

The aim of this study was not to characterize the microbial composition of PFCs per se, but rather to identify virulent organisms associated with poorer clinical outcomes following EUS-guided therapy. Therefore, we included all infected collections irrespective of infection route or timing. *Enterococcus*, *Streptococcus*, and *Candida* species were isolated in 26.9%, 13.1%, and 21.9% of cases, respectively, matching the distribution reported in previous national studies (37%, 16%, and 20%, respectively)^10^. However, the microbial spectrum in infected PFCs may vary considerably across regions, healthcare settings, and patient referral status. For example, a study from India reported Gram-negative bacterial infections in 86% of cases and XDR microorganisms in 25%, substantially higher than in our cohort (30.9% and 1.5%, respectively), while *Enterococcus* species were detected in only 7% of cases^20^. In another study, patients transferred from external institutions exhibited higher rates of polymicrobial infections, as well as infections with *Enterococcus* and *Candida* species, compared with those directly admitted to the reference center^8^.

Fungal microorganisms, which are commonly isolated later in the disease course, were also frequent in our cohort. *Candida albicans* and non-*albicans Candida* species were identified in 21.9% and 12.6% of infected collections, respectively, consistent with previously reported rates of 27.9% and 13.9%^11^. A higher overall fungal infection rate of 46% has been reported in a cohort restricted to patients with walled-off necrosis, which may account for the difference in prevalence. Notably, mortality in that study was comparable to our findings and did not differ substantially by fungal subtype^21^.

Antibiotics are not routinely indicated for sterile collections or pancreatitis without evidence of infection; nevertheless, reported use varies widely and may approach 80% in some healthcare settings^22^. Preprocedural antibiotic exposure is therefore an important potential confounder because it may alter the detectable microbial spectrum without reliably sterilizing an infected collection. Garret et al. showed that antibiotics administered before aspiration failed to sterilize infected collections, even after prolonged broad-spectrum exposure^23^. This may be particularly relevant in late-phase PFCs, where a thick fibrotic capsule and necrotic debris may impair antibiotic penetration and limit the efficacy of systemic therapy.

Conversely, prolonged or inappropriate antibiotic use may promote selection for resistant microorganisms and increase infection complexity. In our cohort, MDR microorganisms were identified in 29.6% of cases, including 4-MRGN (0.8%), 3-MRGN (3.0%), MRSA (0.8%), and VRE (6.5%). Except for VRE, these rates were lower than those reported in another national study (4.9%, 5.7%, 1.6%, and 3.3%, respectively)^11^. To our knowledge, the 26 VRE cases included in our study represent the largest reported cohort to date.

These findings support an individualized antimicrobial strategy. In severely ill patients, empirical antimicrobial therapy may need to include coverage for *Enterococcus* spp. and fungal pathogens. However, antimicrobial stewardship remains essential and should focus on avoiding unnecessary antibiotics in sterile collections, early pathogen identification, susceptibility testing, and timely de-escalation from broad-to narrow-spectrum therapy^24^.

*Enterococcus* infection of pancreatic necrosis has been identified as an independent predictor of mortality and ICU admission in previous studies^25^. The mortality rate in our cohort was lower than in these reports, which may be attributed to the inclusion of only those patients who underwent EUS-guided therapy, typically in the late phase of the disease (median 21 days after symptom onset)^26^. Consequently, early mortality related to the inflammatory phase and organ failure was not captured, potentially selecting for a population with a more favorable baseline prognosis. Moreover, clinical outcomes are likely determined by several factors beyond microbial spectrum and antimicrobial resistance, including adequacy of source control, pancreatic duct disruption, and communication with the gastrointestinal tract.

Collections with comparable amounts of necrosis are associated with worse clinical outcomes in the presence of infection. Endoscopic necrosectomy was required in 30.9% of cases, accounting for a total of 366 procedures during the 12-month follow-up. As expected, additional necrosectomy facilitated resolution and was linked to clinical success in our cohort. However, it remains unclear whether a specific microorganisms—such as *Enterococcus—*directly drives worse outcomes or simply reflects underlying multimorbidity and extensive necrosis, as suggested by our risk factor analysis. Similarly to MDR microorganisms and *Candida* infections, *Enterococcus* species typically emerge later in the disease course and may serve as a surrogate marker of clinical complexity. To address potential confounding, we conducted a sensitivity analysis within the step-up group undergoing necrosectomy, characterized by larger collections, high necrotic content, and pancreatitis as the predominant etiology. In this subgroup, *Enterococcus* infection remained significantly associated with incomplete resolution in multivariable regression analysis (aHR 2.5).

Establishing a causal role for *Enterococcus* spp. in delayed PFC resolution is challenging, as infections are frequently polymicrobial and often occur after broad-spectrum antibiotic exposure. Prior antibiotic therapy may select for *Enterococcus faecium* by suppressing susceptible co-pathogens, and therapeutic options are limited when resistant strains, particularly VRE, are involved. However, resistance alone may not fully explain the pathogenicity of these organisms; additional factors, including cell surface structures, collagen-degrading activity, and biofilm formation, may contribute to persistence and delayed resolution^27^. Although our findings argue against a purely bystander role of VRE, causal inference remains limited by the small sample size. Prospective controlled studies are needed to clarify causality.

Drainage timing, indications, and stent selection require careful interpretation, as our cohort included PFCs irrespective of etiology, consistent with a recent prospective study^28^. In selected patients, collections had been present for longer periods and were drained only after progressive enlargement or symptoms related to mass effect; therefore, shorter intervals from symptom onset to drainage, particularly in non-infected collections, should not be interpreted as routine early drainage of immature post-acute pancreatitis collections. In contrast, delayed endoscopic drainage may have influenced the microbial composition and the observed rate of MDRs in aspirates compared with early radiological sampling.

Although LAMS are increasingly used, most necrotic collections in our cohort were managed with at least three double-pigtail plastic stents to ensure adequate drainage and facilitate repeated necrosectomy sessions. Two randomized trials demonstrated comparable clinical success, hospital stay, and safety between the two approaches, with a shorter procedure time in the LAMS group^29,30^. In our cohort, stent type did not differ between infected and non-infected collections and was not associated with clinical success. Therefore, stent selection should remain individualized according to collection characteristics, indication, and local expertise.

The risk of specimen contamination during FNA cannot be entirely excluded and may have affected the spectrum of microorganisms identified. PFCs may also harbor commensal or low-virulence microbiota whose clinical relevance to disease progression warrants further investigation^31,32^. Although culture-based microbiology remains essential for susceptibility-guided therapy, it may underestimate microbiome complexity in PFCs, particularly in polymicrobial infections, after prior antibiotic exposure, or when difficult-to-culture microorganisms are present^33^. External validation in cohorts using next-generation sequencing and standardized molecular pipelines would enable more comprehensive microbiological stratification of aspirated fluid compared with culture-based approaches and would represent a promising area for prospective studies.

Despite the inclusion of nearly 400 patients, several limitations should be acknowledged. First, although the multicenter design enhances external validity, center-specific differences in endoscopic techniques and heterogeneity in PFC etiology may have influenced resolution rates and clinical outcomes. Second, patients with previous PFC drainage were excluded, as prior intervention may substantially affect both subsequent resolution and microbiological findings; while justified, this criterion may introduce selection bias and limit generalizability. Third, as this was a real-world cohort, microbiological sampling was not obtained in all cases, particularly when patients did not meet clinical criteria suggestive of infection and drainage was performed solely for mass effect. Fourth, prior antibiotic exposure may have influenced microbial composition and resistance patterns and should therefore be considered a potential confounder. Moreover, imaging signs of infection were incorporated into clinical decision-making but were not systematically captured as discrete variables, potentially introducing misclassification bias. Nevertheless, EUS-guided sampling was performed whenever infection was clinically suspected, and microbiological confirmation was considered diagnostic in such cases.

In conclusion, this study demonstrated that the microbiological spectrum and antimicrobial resistance were independently associated with outcomes following EUS-guided drainage of PFCs beyond morphological criteria^4^, identifying Enterococcus species—particularly vancomycin-resistant strains and polymicrobial resistant pathogens—as key predictors of treatment failure. In high-risk scenarios, a more intensified endoscopic approach, including early complete debridement to enhance source control and limit complications, should be considered. These patients may also require closer surveillance and timely referral to pancreatic centers with established expertise in interventional endoscopy, interventional radiology, and pancreatic surgery. A microbiology-based risk stratification framework and a shift toward precision-guided management of PFCs may substantially improve the current standard of care.

**Table 4:** Multivariable Cox regression analysis of risk factors for incomplete resolution of pancreatic fluid collections on follow-up imaging, including a sensitivity analysis in the subgroup of patients who underwent necrosectomy.

Variables	All PFCs		PFCs with Necrosectomy	
	aHR (95% CI)	*p*-value	aHR (95% CI)	*p*-value
Male sex	1.32 (1.00–1.69)	0.06	1.08 (0.60–1.92)	0.82
Size > 8 cm	1.33 (1.00–1.79)	0.05	2.33 (1.30–4.17)	0.005
Etiology	1.37 (1.03–1.82)	0.03	0.97 (0.47–2.04)	0.94
Presence of necrosis	1.14 (0.89–1.52)	0.37	0.63 (0.27–1.46)	0.28
DPS	0.88 (0.60–1.30)	0.53	0.49 (0.25–0.96)	0.04
Multiple stents	0.89 (0.68–1.18)	0.42	0.41 (0.23–0.74)	0.003
IAT	1.18 (0.80–1.75)	0.41	1.46 (0.77–2.75)	0.25
Gram-positive	1.41 (0.88–2.22)	0.15	2.08 (0.89–5.00)	0.09
*Enterococcus spp.*	1.47 (1.03–2.13)	0.03	2.50 (1.39–4.55)	0.002
Multiple MDR	1.56 (0.96–2.56)	0.07	1.27 (0.64–2.44)	0.51
*Candida*, non-albicans	1.23 (0.81–1.85)	0.33	1.61 (0.88–2.94)	0.12

Abbreviations: DPS, double-pigtail plastic stents; aHR, adjusted hazard ratio; IAT, inadequate antimicrobial therapy; MDR, multidrug-resistant; PFC, pancreatic fluid collection.

**Table 5:** Cox regression analysis of risk factors for incomplete resolution of acute pancreatitis-associated fluid collections with available microbiology data (*n* = 217).

Variables	Univariable		Multivariable^a^	
Patient characteristics	HR (95% CI)	*p*-value	HR (95% CI)	*p*-value
Age ≥ 65 years	1.05 (0.68–1.62)	0.83		
Male sex	1.07 (0.74–1.54)	0.74		
BMI ≥ 30 kg/m²	0.56 (0.34–0.91)	0.02	0.63 (0.30–1.33)	0.23
Comorbidity	1.20 (0.83–1.73)	0.34		
ASA ≥ 3	1.30 (0.93–1.81)	0.12		
Pre-drainage organ failure	1.24 (0.79–1.96)	0.35		
Severe acute pancreatitis	1.13 (0.79–1.63)	0.51		
**Collection characteristics**				
Size > 8 cm	2.36 (1.67–3.33)	< 0.01	3.94 (2.19–7.07)	< 0.01
Necrosis extent > 30%	1.33 (0.81–2.17)	0.26		
**Therapy**				
DPS	0.71 (0.45–1.11)	0.14		
Multiple stents	0.92 (0.66–1.29)	0.64		
Percutaneous drainage	1.23 (0.86–1.76)	0.26		
Immunosuppressive therapy	1.37 (0.74–2.52)	0.32		
Previous antibiotic therapy	1.38 (0.93–2.05)	0.12		
IAT	1.32 (0.84–2.08)	0.23		
**Infection characteristics**				
Gram-positive	1.55 (1.11–2.16)	< 0.01	1.28 (0.75–2.17)	0.36
Gram-negative	1.15 (0.81–1.63)	0.44		
Polymicrobial	1.33 (0.96–1.85)	0.08	1.01 (0.60–1.72)	0.96
*Enterococcus spp.*	1.79 (1.23–2.59)	< 0.01	1.69 (1.12–2.77)	0.01
VRE	1.45 (0.84–2.53)	0.18		
MDR	1.35 (0.95–1.91)	0.09	1.21 (0.73–1.98)	0.46
Multiple MDR	2.12 (1.14–3.92)	0.01	1.85 (0.88–3.86)	0.10
XDR	1.94 (0.27–13.77)	0.50		
MRGN	1.18 (0.58–2.41)	0.64		
*Candida albicans*	0.96 (0.66–1.39)	0.82		
*Candida*,* non-albicans*	1.55 (0.96–2.52)	0.07	1.11 (0.59–2.08)	0.74

Abbreviations: ASA, American Society of Anesthesiologists; BMI, body mass index; DPS, double-pigtail plastic stents; HR, hazard ratio; IAT, inadequate antimicrobial therapy; MDR, multidrug-resistant; MRGN, multi resistant Gram-negative bacteria; PFC, pancreatic fluid collection; VRE, vancomycin-resistant Enterococcus; XDR, extensively drug-resistant. ^a^Multivariable model adjusted for the following covariates: age, sex, comorbidity, PFC size, and extent of necrosis.

## Supplementary Information

Below is the link to the electronic supplementary material.

Supplementary Material 1

## Data Availability

The data and study materials are available from the corresponding author upon reasonable request.
