# Supplementary material for: Microbial spectrum and antimicrobial resistance are associated with outcomes after endoscopic ultrasound-guided therapy of pancreatic fluid collections
Source: Sci Rep. 2026 Aug 5;16:24152. doi: 10.1038/s41598-026-64436-5 (PMC13443581; doi:10.1038/s41598-026-64436-5)
Supplement: Supplementary file 1 [file 41598_2026_64436_MOESM1_ESM.docx]

**Microbial Spectrum and Antimicrobial Resistance Are Associated with Outcomes After Endoscopic Ultrasound-Guided Therapy of Pancreatic Fluid Collections**

**Marcel Razpotnik^1,*^, Christian Jürgensen^1^, Michael Sigal^1^, Toni Herta^1^, Özlem Çelebi^1^, Andreas Adler^1^, Silke Leonhardt^1^, Federica Branchi^2^, Juliane Buchkremer^2^, Konstantinos Kouladouros^1^, Alexander Wree^1,3^, Jürgen Pilz^4^, Markus M. Heimesaat^5^, Britta Siegmund^2^, Cornelius Engelmann^1^, Frank Tacke^1^**

^1^Department of Hepatology and Gastroenterology, Campus Virchow-Klinikum (CVK) and Campus Charité Mitte (CCM), Charité - Universitätsmedizin Berlin, Germany

^2^Department of Gastroenterology, Infectology and Rheumatology, Campus Benjamin Franklin (CBF), Charité - Universitätsmedizin Berlin, Germany

^3^Department of Gastroenterology, Vivantes Hospital Friedrichshain Berlin, Berlin, Germany

^4^Department of Statistics, University of Klagenfurt, Klagenfurt, Austria

^5^Institute of Microbiology, Infectious Diseases and Immunology, Charité - Universitätsmedizin Berlin, Corporate Member of Freie Universität Berlin, Humboldt-Universität zu Berlin, and Berlin Institute of Health, Berlin, Germany

***Supplementary Material***

**METHODS**

**Supplementary Figure 1.** Study flow-chart.

Abbreviations: CDS, Choledochoduodenostomy; EUS, Endoscopic Ultrasound; GBD, Gallbladder Drainage; HGS, Hepaticogastrostomy; PDD, Pancreatic Duct Drainage; PFC, Pancreatic Fluid Collection.

**Microbiological assessment**

Initial evaluation included Gram staining and direct microscopy, followed by aerobic and anaerobic cultures on selective and non-selective media. Bacteria were further classified according to their antibiotic susceptibility profile as defined by the European Committee for Antimicrobial Susceptibility Testing (EUCAST). Multidrug-resistant (MDR) bacteria were defined as having acquired non-susceptibility to ≥1 agent in ≥3 antimicrobial categories; extensively drug-resistant (XDR) bacteria retained susceptibility to ≤2 antimicrobial categories; and pandrug-resistant (PDR) bacteria exhibited resistance to all agents in all antimicrobial categories for which susceptibility testing was performed[15].

**RESULTS**

**Supplementary Table 1.** Median number of antimicrobial agents and cumulative duration of therapy during hospitalization.

| **Antimicrobial Class** | **Patients** | **Co-Antibiotics^a^** | **Duration^b^** |
| --- | --- | --- | --- |
|  | **% (N)** | **N (IQR)** | **days (IQR)** |
| Aminoglycosides | 2.3 (9) | 8 (5.5-10) | 70 (36.8-87.8) |
| Carbapenems | 38.2 (152) | 3 (2-5) | 20 (12-39.3) |
| Caspofungin | 14.8 (59) | 6 (4-7) | 31 (15-45) |
| Cephalosporins | 20.4 (81) | 3 (2-6) | 15 (10-35.5) |
| Fluconazole | 4.3 (17) | 5 (4-6) | 24.5 (16-36) |
| Linezolid | 8.0 (32) | 4.5 (3-6.5) | 35 (15.8-45.8) |
| Macrolides | 0.8 (3) | 10 (6.3-10) | 93 (55.5-127) |
| Nitroimidazoles | 12.8 (51) | 3 (2-5) | 13 (9.3-29.3) |
| Penicillin derivatives | 42.0 (167) | 2 (1-5) | 15 (10-32) |
| Quinolones | 12.6 (50) | 2 (2-6) | 24 (10-48) |
| Tigecycline | 4.3 (17) | 6 (3-8) | 56 (28-89.5) |
| Vancomycin | 21.4 (85) | 4 (2.8-6) | 26.5 (15-48) |
| Others | 7.3 (29) | 5 (2-8) | 35 (14.8-63.8) |

Abbreviations: IQR, interquartile range; N, number. ^a^Median number of antibiotics administered during hospitalization, either concurrently or sequentially; ^b^Median cumulative duration of antibiotic therapy during hospitalization.

**Supplementary Table 2.** Incidence of the most common microorganisms isolated from infected pancreatic fluid collections.

| **Gram-positive bacteria** | **% (N)** | **Gram-negative bacteria (cont.)** | **% (N)** |
| --- | --- | --- | --- |
| *Actinomyces* spp. | 1.0 (4) | *Klebsiella pneumoniae* | 7.3 (29) |
| *Enterococcus* spp. | 26.9 (107) | *Klebsiella oxytoca* | 1.5 (6) |
| *Enterococcus faecalis* | 8.8 (35) | *Pseudomonas* spp. | 3.8 (15) |
| *Enterococcus faecium* | 18.8 (75) | *Pseudomonas aeruginosa* | 3.0 (12) |
| *Enterococcus*, others | 0.5 (2) | *Prevotella* spp. | 4.0 (16) |
| *Staphylococcus* spp. | 9.8 (39) | *Prevotella buccae* | 1.8 (7) |
| *Staphylococcus epidermidis* | 7.5 (30) | *Prevotella melaninogenica* | 1.5 (6) |
| *Staphylococcus aureus* | 2.5 (10) | *Prevotella denticola* | 0.8 (3) |
| *Staphylococcus haemolyticus* | 2.3 (9) | *Proteus* spp. | 1.5 (6) |
| *Staphylococcus hominis* | 0.5 (2) | *Proteus mirabilis* | 1.5 (5) |
| *Staphylococcus warneri* | 0.5 (2) | *Serratia* spp. | 2.3 (9) |
| *Streptococcus* spp. | 13.1 (52) | *Serratia marcescens* | 1.8 (7) |
| *Streptococcus mitis/ oralis* | 3.3 (13) | *Veillonella* spp. | 1.5 (5) |
| *Streptococcus parasanguinis* | 2.3 (9) | *Veillonella atypica* | 0.8 (3) |
| *Streptococcus anginosus* | 1.8 (7) |  |  |
| *Streptococcus salivarius* | 1.5 (6) | **Candida species** | **% (N)** |
| *Streptococcus constellatus* | 1.5 (6) | *Candida albicans* | 21.9 (87) |
| *Lactobacillus* spp. | 2.8 (11) | *Candida krusei* | 2 (8) |
|  |  | *Candida dubliniensis* | 0.8 (3) |
| **Gram-negative bacteria** | **% (N)** | *Candida glabrata* | 6.3 (25) |
| *Acinetobacter baumannii* | 0.5 (2) | *Candida tropicalis* | 12.6 (5) |
| *Bacteroides* spp. | 3.3 (13) | *Candida*, others | 2.3 (9) |
| *Bacteroides fragilis* | 1.8 (7) |  |  |
| *Bacteroides vulgatus* | 0.5 (2) | **Resistant microorganisms** | **% (N)** |
| *Citrobacter* spp. | 3.0 (12) | MRSA | 0.8 (3) |
| *Citrobacter freundii* | 1.3 (5) | VRE | 6.5 (26) |
| *Citrobacter koseri* | 1.0 (4) | 3-MRGN | 3.0 (12) |
| *Escherichia coli* | 8.3 (33) | 4-MRGN | 0.8 (3) |
| *Enterobacter cloacae* | 4.8 (19) | MDR | 29.6 (118) |
| *Fusobacterium nucleatum* | 0.5 (2) | XDR | 1.5 (6) |
| *Klebsiella* spp. | 9.0 (36) | PDR | 0.3 (1) |

Abbreviations: MDR, multidrug-resistant; MRGN, multiresistant Gram-negative bacteria; MRSA, methicillin-resistant *Staphylococcus aureus*; N, number; PDR, pandrug-resistant; VRE, Vancomycin-resistant *Enterococcus*; XDR, extensively drug-resistant.

Only microorganisms identified in at least two cases are presented in the table. The following microorganisms were isolated in a single case: *Abiotrophia defective, Actinomyces graevenitzii, Actinomyces israelii, Alloscardovia omicolens, Bacillus cereus, Bacteroides dorei, Bacteroides ovatus, Bacteroides thetaiotaomicron, Bacteroides isolvens, Clostridium clostridioforme, Collinsella aerofaciens, Corynebacterium amycolatum, Cutibacterium acnes, Eikenella corrodens, Enterococcus gallinarum, Enterococcus hirae, Klebsiella aerogenes, Morganella morganii, Neisseria subflava, Ochrobactrum intermedium, Pandoraea sputorum, Parvimonas micra, Proteus vulgaris, Providencia stuartii, Pseudomonas alcaligenes, Pseudomonas oryzihabitans, Pseudomonas stutzeri, Rahnella aquatilis, Raoultella ornithinolytica, Rothia dentocariosa, Serratia liquefaciens, Serratia odorifera, Staphylococcus capitis, Staphylococcus caprae, Streptococcus viridans, Veillonella dispar, Veillonella parvula.*

**Supplementary Table 3.** Sensitivity analysis: Cox regression predictors of incomplete resolution, restricted to patients with infected pancreatic walled-off necrosis (n=118).

| Variables | **Univariable** | | **Multivariable^a^** | |
| --- | --- | --- | --- | --- |
| **Patient** | **HR (95% CI)** | ***p*-value** | **HR (95% CI)** | ***p*-value** |
| Age ≥ 65 years | 0.91 (0.50–1.66) | 0.75 |  |  |
| Male | 1.22 (0.71–2.11) | 0.47 |  |  |
| BMI ≥ 30 kg/m² | 0.50 (0.25–1.00) | 0.05 | 0.62 (0.29–1.36) | 0.24 |
| Comorbidity | 1.41 (0.85–2.34) | 0.18 |  |  |
| ASA ≥ 3 | 1.09 (0.69–1.73) | 0.71 |  |  |
| Pre-drainage organ failure | 0.90 (0.50–1.65) | 0.75 |  |  |
| **Collection** |  |  |  |  |
| Size > 8 cm | 2.77 (1.68–4.57) | <0.001 | 2.77 (1.54–4.97) | <0.001 |
| Etiology | 1.60 (0.93–2.77) | 0.09 | 1.20 (0.63–2.26) | 0.58 |
| Necrosis extent > 30% | 1.25 (0.77–2.03) | 0.37 |  |  |
| **Therapy** |  |  |  |  |
| DPS | 0.45 (0.19–1.03) | 0.06 | 0.58 (0.24–1.38) | 0.22 |
| Multiple stents | 0.99 (0.61–1.61) | 0.98 |  |  |
| Percutaneous drainage | 0.97 (0.60–1.56) | 0.90 |  |  |
| Immunosuppressive therapy | 1.25 (0.39–3.96) | 0.71 |  |  |
| Previous antibiotic therapy | 1.91 (0.95–3.86) | 0.09 |  |  |
| IAT | 1.33 (0.79–2.25) | 0.28 |  |  |
| **Infection** |  |  |  |  |
| Gram-positive | 2.69 (1.34–5.38) | 0.005 | 2.33 (1.08–5.04) | 0.03 |
| Gram-negative | 0.80 (0.50–1.27) | 0.34 |  |  |
| Polymicrobial | 1.10 (0.48–2.54) | 0.82 |  |  |
| *Enterococcus spp.* | 2.34 (1.45–3.77) | <0.001 | 2.24 (1.37–3.67) | 0.001 |
| VRE | 1.25 (0.67–2.32) | 0.49 |  |  |
| MDR | 1.18 (0.72–1.94) | 0.51 |  |  |
| MDR multiple | 1.63 (0.90–2.98) | 0.11 | 1.62 (0.86–3.05) | 0.13 |
| XDR | 0.69 (0.22–2.17) | 0.53 |  |  |
| MRGN | 1.17 (0.50–2.72) | 0.72 |  |  |
| *Candida albicans* | 0.62 (0.38–1.00) | 0.05 | 0.63 (0.38–1.04) | 0.07 |
| *Candida, non-albicans* | 1.20 (0.72–1.98) | 0.49 |  |  |

Abbreviations: ASA, American Society of Anesthesiologists; BMI, body mass index; DPS, double-pigtail plastic stents; HR, hazard ratio; IAT, inadequate antimicrobial therapy; MDR, multidrug-resistant; MRGN, multi resistant Gram-negative bacteria; PFC, pancreatic fluid collection; VRE, Vancomycin-resistant Enterococcus; XDR, extensively drug-resistant. ^a^Multivariable model adjusted for the following covariates: age, gender, comorbidity, PFC size, and etiology.

**Supplementary Table 4.** Multinomial logistic regression identifying risk factors for Enterococcus- and non-Enterococcus-infected PFCs versus sterile controls.

| Variables | **Enterococcus *vs.* Control** | | **Non-Enterococcus *vs.* Control** | |
| --- | --- | --- | --- | --- |
|  | **RRR (95% CI)** | ***p*-value** | **RRR (95% CI)** | ***p*-value** |
| **Patient** |  |  |  |  |
| Age ≥ 65 years | 0.93 (0.54-1.59) | 0.79 | 0.48 (0.27-0.86) | 0.01 |
| Male | 1.91 (1.10-3.32) | 0.02 | 1.48 (0.89-2.45) | 0.13 |
| BMI ≥ 30 kg/m² | 0.94 (0.46-1.94) | 0.88 | 0.86 (0.43-1.71) | 0.67 |
| Comorbidity | 1.92 (1.08-3.42) | 0.03 | 1.16 (0.70-1.92) | 0.57 |
| ASA ≥ 3 | 2.72 (1.64-4.53) | <0.001 | 1.11 (0.70-1.77) | 0.66 |
| Pre-drainage organ failure | 1.35 (0.73-2.50) | 0.34 | 1.00 (0.54-1.85) | 0.99 |
| **Collection** |  |  |  |  |
| Size > 8 cm | 4.39 (2.51-7.68) | <0.001 | 1.58 (0.98-2.56) | 0.06 |
| Etiology | 1.93 (1.16-3.23) | 0.01 | 1.69 (1.04-2.74) | 0.03 |
| Presence of necrosis | 4.98 (2.95-8.45) | <0.001 | 1.62 (0.99-2.66) | 0.05 |
| Necrosis extent > 30% | 2.26 (0.98-5.25) | 0.06 | 0.56 (0.21-1.46) | 0.23 |
| **Therapy** |  |  |  |  |
| DPS | 0.97 (0.50-1.90) | 0.94 | 4.30 (1.60-11.6) | 0.004 |
| Multiple stents | 2.45 (1.45-4.14) | <0.001 | 2.65 (1.60-4.41) | <0.001 |
| Necrosectomy | 5.0 (2.88-8.67) | <0.001 | 2.14 (1.24–3.72) | 0.007 |
| Percutaneous drainage | 4.11 (2.09-8.07) | <0.001 | 1.17 (0.54-2.55) | 0.70 |
| Immunosuppressive therapy | 0.81 (0.33–1.99) | 0.65 | 1.27 (0.59–2.74) | 0.54 |
| Previous antibiotic therapy | 2.02 (1.03-3.97) | 0.04 | 1.30 (0.74-2.29) | 0.37 |
| IAT | 3.49 (1.10-11.0) | 0.03 | 1.10 (0.34-3.59) | 0.88 |

Abbreviations: ASA, American Society of Anesthesiologists; BMI, body mass index; CI, confidence interval; DPS, double-pigtail plastic stents; IAT, inadequate antimicrobial therapy; PFC, pancreatic fluid collection; RRR, relative risk ratio.

**Supplementary Table 5.** Clinical characteristics in PFC patients with vs. without direct endoscopic necrosectomy.

| Variables | **PFCs with Necrosectomy** | **PFCs without Necrosectomy** | ***p*-value^a^** |
| --- | --- | --- | --- |
|  | (n=123) | (n=275) |  |
| **Patient** |  |  |  |
| Age ≥ 65 years (%) | 26 (21.1) | 74 (26.9) | 0.40 |
| Male (%) | 93 (75.6) | 188 (68.4) | 0.60 |
| BMI ≥ 30 kg/m² (%) | 14 (11.4) | 41 (14.9) | 0.50 |
| Comorbidity (%) | 92 (74.8) | 196 (71.3) | 0.84 |
| ASA ≥ 3 (%) | 58 (47.2) | 142 (51.6) | 0.70 |
| Pre-drainage organ failure | 21 (17.1) | 51 (18.5) | 0.88 |
| **Collection** |  |  |  |
| Size > 8 cm (%) | 94 (76.4) | 104 (37.8) | <0.001 |
| Etiology (%) | 105 (85.4) | 145 (52.7) | 0.005 |
| Presence of necrosis (%) | 106 (86.2) | 58 (21.1) | <0.001 |
| Necrosis extent > 30% (%) | 43 (35.0) | 18 (6.5) | <0.001 |
| **Therapy** |  |  |  |
| DPS (%) | 102 (82.9) | 241 (87.6) | 0.79 |
| Multiple stents (%) | 87 (70.7) | 55 (20) | <0.001 |
| Percutaneous drainage (%) | 24 (19.5) | 35 (12.7) | 0.18 |
| Immunosuppressive therapy | 7 (5.7) | 30 (10.9) | 0.18 |
| Previous antibiotic therapy (%) | 114 (92.7) | 164 (59.6) | 0.009 |
| IAT (%) | 33 (26.8) | 37 (13.5) | 0.01 |
| **Infection** |  |  |  |
| Infection (%) | 94 (76.4) | 138 (50.2) | 0.02 |
| Gram-positive (%) | 81 (65.9) | 100 (36.4) | 0.001 |
| Gram-negative (%) | 50 (40.7) | 73 (26.5) | 0.06 |
| Polymicrobial (%) | 84 (68.3) | 114 (41.5) | 0.007 |
| *Enterococcus spp.* (%) | 55 (44.7) | 52 (18.9) | <0.001 |
| VRE (%) | 20 (16.3) | 6 (2.2) | <0.001 |
| MDR (%) | 56 (45.5) | 62 (22.5) | 0.001 |
| MDR multiple (%) | 23 (18.7) | 15 (5.5) | <0.001 |
| XDR (%) | 3 (2.4) | 3 (1.1) | 0.58 |
| MRGN (%) | 10 (8.1) | 5 (1.8) | 0.009 |
| *Candida albicans* (%) | 34 (27.6) | 53 (19.3) | 0.18 |
| *Candida, non-albicans* (%) | 29 (23.6) | 21 (7.6) | <0.001 |

Abbreviations: ASA, American Society of Anesthesiologists; BMI, body mass index; DPS, double-pigtail plastic stents; HR, hazard ratio; IAT, inadequate antimicrobial therapy; MDR, multidrug-resistant; MRGN, multi resistant Gram-negative bacteria; PFC, pancreatic fluid collection; VRE, Vancomycin-resistant Enterococcus; XDR, extensively drug-resistant. ^a^Chi-square Test.
